# Application of light detection and ranging and ultrasonic sensors to high-throughput phenotyping and precision horticulture: current status and challenges

**DOI:** 10.1038/s41438-018-0043-0

**Published:** 2018-07-01

**Authors:** André F. Colaço, José P. Molin, Joan R. Rosell-Polo, Alexandre Escolà

**Affiliations:** 1 Biosystems Engineering Department, “Luiz de Queiroz” College of Agriculture, University of São Paulo, Av. Pádua Dias, 11, Piracicaba - SP 13418-900 Brazil; 2 Research Group on AgroICT and Precision Agriculture, Department of Agricultural and Forest Engineering, School of Agrifood and Forestry Science and Engineering, University of Lleida – Agrotecnio Center, Av. Rovira Roure, 191, 25198 Lleida, Catalonia Spain; 3 Present Address: CSIRO, Waite Campus, Locked Bag 2, Glen Osmond, SA 5064 Australia

## Abstract

Ultrasonic and light detection and ranging (LiDAR) sensors have been some of the most deeply investigated sensing technologies within the scope of digital horticulture. They can accurately estimate geometrical and structural parameters of the tree canopies providing input information for high-throughput phenotyping and precision horticulture. A review was conducted in order to describe how these technologies evolved and identify the main investigated topics, applications, and key points for future investigations in horticulture science. Most research efforts have been focused on the development of data acquisition systems, data processing, and high-resolution 3D modeling to derive structural tree parameters such as canopy volume and leaf area. Reported applications of such sensors for precision horticulture were restricted to real-time variable-rate solutions where ultrasonic or LiDAR sensors were tested to adjust plant protection product or fertilizer dose rates according to the tree volume variability. More studies exploring other applications in site-specific management are encouraged; some that integrates canopy sensing data with other sources of information collected at the within-grove scale (e.g., digital elevation models, soil type maps, historical yield maps, etc.). Highly accurate 3D tree models derived from LiDAR scanning demonstrate their great potential for tree phenotyping. However, the technology has not been widely adopted by researchers to evaluate the performance of new plant varieties or the outcomes from different management practices. Commercial solutions for tree scanning of whole groves, orchards, and nurseries would promote such adoption and facilitate more applied research in plant phenotyping and precision horticulture.

## Remote sensing: Shining light on tree productivity

LIDAR should be combined with other information from groves or orchards to gain a better understanding of the factors driving tree performance and optimize it. LIDAR is a remote sensing method which ‘maps’ surfaces by shining light at them and measuring how long the light takes to return. Within horticulture, it’s main use has been in estimating canopy volume to optimize spraying of eg. fruit trees, or fertilizer distribution. In this review, André Colaço at the University of São Paulo in Brazil and colleagues describe the evolution of this technology and its broader applications. They suggest that by combining LIDAR with other information, such as soil electrical conductivity and fertility; or historic yield, disease and pest occurrence, growers could fine-tune crop production. LIDAR could also be used to evaluate the performance of new plant varieties.

## Introduction

Collecting information over a grove or orchard has been greatly facilitated in the past few decades with the development of different types of sensors within the scope digital horticulture. Digital horticulture is a recent terminology that refers to the use of a range of digital technologies (including plant sensing devices) used in different horticultural applications such as the high-throughput phenotyping and precision horticulture (often referred as site-specific management).

Among different types of sensing technologies applied in digital horticulture, ranging sensors, mostly light detection and ranging (LiDAR) and ultrasonic sensors, gained attention from researchers and practitioners for their applications in fruit and nut crops. Ranging sensors are designed to measure the distance to the nearest object by emitting an electromagnetic signal (an ultrasonic wave for ultrasonic sensors or a laser beam for LiDAR sensors) in a given direction; the time between emitting and receiving the signal is used to calculate distance to the target. As long as appropriate acquisition and data processing is applied, these sensors can be used to estimate geometrical parameters such as canopy height, width, volume, and other structural parameters. These parameters are useful to site-specific management because they usually relate to plant development, health, yield potential, and, consequently, with input requirements. With such information, growers can identify zones with different characteristics within the grove/orchard and apply appropriate management in each zone. If the data are provided with sufficient spatial resolution, trees can be treated individually using automated variable rate application of inputs.

Besides its applications at a grove/orchard management level, LiDAR sensors have also been regarded as effective tools for high-throughput phenotyping, given that plant architecture, growth, and other structural characteristics of the trees are important components of plant phenomics. Due to their capability to rapidly and objectively estimate relevant tree parameters with high accuracy and precision, LiDAR sensors are an alternative to laborious traditional methods used in basic horticulture research (e.g., plant breeding).

Whilst other reviews^1–7^ covered aspects of these sensors in different contexts or with different focus, this literature review describes how ultrasonic and LiDAR sensors applied to high-throughput phenotyping and precision horticulture evolved since the earliest studies and identifies which subjects have gained more attention from researchers and which are still lacking in research. This will be achieved by means of a narrative over the developments, followed by a discussion where some key points for future investigation will be highlighted.

## Review scope

**Fig. 1:**
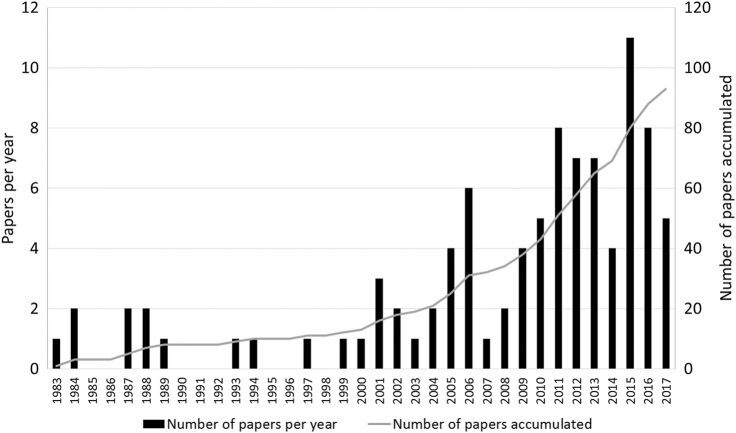
Publication rate of reviewed studies

A search for studies on LiDAR and ultrasonic sensors applied to tree crops (excluding forestry) was carried out using the databases of scientific publication that gather the most relevant journals in the fields of agricultural and horticultural science. Over 90 papers were selected and analyzed (Fig. 1). Publication dates ranged from 1983 until 2017. About 70% of this material was published in the past decade.

The particular topic of this research relates to many different fields of science. Some areas worth mentioning are: horticulture, including soil fertility and plant protection management, plant phenomics, site-specific management, remote sensing and instrumentation, machine automation, and computer modeling. Forestry science had an important role in the development of LiDAR as a remote sensing technology and might be taken as an inspiration to the LiDAR studies in horticulture. However, the focus of this review remained in horticultural applications of these sensors. Applications of LiDAR sensors as guidance for autonomous vehicle were not covered either.

Three main research groups were identified as important contributors to the theme of ultrasonic and LiDAR sensors applied to horticultural tree crops. Researchers from Catalonia, Spain, are responsible for the most recent studies. They stand out with studies dedicated to LiDAR applications to several fruit crops, especially apple, olive, and vineyards. They might be considered focused on the LiDAR technology itself and not on a specific crop. The second group, from Florida, USA, is focused on the development of sensors specifically for citrus. Most of their work employed ultrasonic sensors, although some important studies with LiDAR sensors were also conducted. Studies from UK are more dedicated to the spraying technology applied mostly to apple orchards. This group is responsible for presenting some of the early approaches of LiDAR sensors applied to fruit tree crops.

## Early developments and real-time variable rate application of inputs

**Fig. 2:**
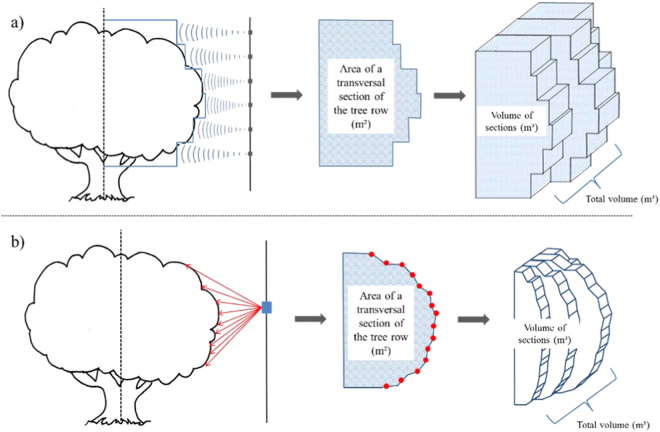
Measurements of canopy volume by ranging sensors. Example of ultrasonic (**a**) and LiDAR (**b**) sensors

The canopy volume of tree row crops can be estimated by different methods. One that is often used to calculate spraying dose rates is based on the tree-row-volume concept^8–11^ which traditionally uses manual measurements of canopy height, width, and length to calculate volume. Early studies on ranging sensors applied to tree crops aimed to make such measurements more accurate and rapid. In the studies of McConnell et al.^12^ in West Virginia and Giles et al.^13–15^ in California, USA, ultrasonic sensing systems were designed and evaluated. The sensors were arranged in different heights along a vertical pole, facing a side of the tree row. Each ultrasonic unit measured its distance to the canopy as the system moved along the alleyway at constant speed. By combining the measured distances from the sensors, the system provided estimates of canopy volume for each section along the tree row (Fig. 2a). The authors found it to be an effective and reliable way to measure the canopy volume of fruit trees. It overcame the difficulty of measuring tree size with traditional methods and offered the possibility to optimize spraying application in non-uniform orchards. This principle was later implemented in many ultrasonic measurements of fruit crops^16–20^.

Similarly to early developments of ultrasonic sensors, a sprayer controlling system based on a terrestrial LiDAR scanner was presented in USA in the mid-1990s^21,22^. The proposed set up of the laser sensor, facing the side of the tree vertically and moving along the grove alleys to measure transversal sections of the row, was similar to the ultrasonic systems proposed by McConnell et al.^12^ and by Giles et al.^13–15^ and was later used by most of the studies in the application of LiDAR to tree crops^23,24^. Unlike the ultrasonic sensors, the 2D LiDAR scanner can measure distances in several directions within a plane (Fig. 2b), providing a much more accurate profile of the target. In the UK, Walklate et al.^25,26^ also presented some of the early approaches of a 2D LiDAR scanner applied to fruit tree crops. In their work, the transmission and interception characteristics of the laser beam through the vegetation were evaluated as indicators of the canopy density and spraying deposition. Later on, these authors were able to assess canopy size variability and highlight the poor efficiency of constant flow rate spraying for apple trees of different sizes^27–30^. This initiative looked towards a coordinate adjustment on the label-recommended dose rate of agrochemicals in UK fruit orchards based on tree size and density^31–34^.

Parallel to the UK developments, the ranging sensor technology gained attention from the citrus industry in North America. In early developments, the application of ranging sensors to citrus was mentioned by Whitney et al.^35^ in Florida, USA. This paper presented the main topics that should be developed in order to advance the use of precision agriculture in citrus. The canopy volume and height estimations by ranging sensors were mentioned among yield mapping, variable rate technology, GPS (Global Positioning Systems) and GIS (Geographic Information Systems) topics. Later on, the investigation of both LiDAR and ultrasonic sensors evolved simultaneously in Florida. Schuman and Zaman^36^, Zaman and Salyani^37^, and Zaman and Schumann^38^ evaluated several aspects of ultrasonic estimations of citrus canopy volume. Some of their findings were that ultrasonic measurements were highly correlated with manual measurements (*R*^2^ > 0.90); canopy volume varied significantly within commercial groves; the sensor readings were stable even in different ground speeds; measurements of canopy volume were more reliable in densely foliated trees; and the trees were not symmetrical, so they should be scanned from both sides. The works by Zaman et al.^39^ and Schumann et al.^40^ found a high correlation (*R*^2^ of 0.80 and 0.64, respectively) between canopy volume measured by ultrasonic sensors and fruit yield in commercial citrus plots. Given the potential application of these sensors to site-specific management, Schumann et al.^41^ adapted a fertilizer spreader machine with a control system in order to perform variable rate application based on a single tree prescription map. Zaman et al.^42^ reported up to 40% savings of nitrogen by using this practice compared to fixed rate fertilization.

One work which compared ultrasonic and laser sensors in the Florida citrus was carried out by Tumbo et al.^43^. They implemented a laser measuring system and an ultrasonic system with twenty sensors and compared their capability to estimate canopy volume. The methods correlated well with each other (*R*^2^ = 0.90) and with manual measurements of canopy volume. However, the authors concluded that, due to the higher resolution of laser sensor, it could provide better estimations of canopy volume especially in groves with small replants. The laser sensor was subsequently investigated with greater depth by other authors. Wei and Salyani^44,45^ estimated volume and canopy density by transforming the laser data into 2D distance images, similarly to a previous study by Tumbo et al.^43^. High accuracy and repeatability of the distance measurements by the sensor was reported. Their approach resulted in an average error of 4.4% on the volume estimation of a template box^44^. Lee and Ehsani^46,47^ also analyzed several performance aspects of two commercial laser sensors and proposed a data acquisition and processing method (similar to example shown in Fig. 2b) for quantifying tree height, width, canopy surface area, and volume. They reported an error of 5.9% against manual measurements of canopy volume^47^.

Whilst research in Florida significantly advanced the ranging sensor technology, similar studies were conducted in Catalonia, Spain, but mostly with other tree crops besides citrus. Several studies have developed and demonstrated ultrasonic-based ranging systems for spraying control in different tree crops such as olive, pear and apple groves^19,23,48^, and vineyards^49–51^. Spraying control systems based on LiDAR sensor were also reported by Escolà et al.^23^ and Llorens et al^52^. The outcomes from the validation of sensor-based controlling systems are presented in Table 1.

**Table 1:** Reports of input savings from the use of ultrasonic sensors to control real-time variable rate application of inputs

Reference	Operation	Tree crop	Input savings
Giles et al.^13,15^	Spraying	Peach	28%
Giles et al.^13,15^	Spraying	Apple	52%
Moltó et al.^17^	Spraying	Citrus	30%
Moltó et al.^16^	Spraying	Citrus	37%
Zaman et al.^42^	Fertilizing	Citrus	40%
Solanelles et al.^48^	Spraying	Olive	70%
Solanelles et al.^48^	Spraying	Pear	28%
Solanelles et al.^48^	Spraying	Apple	39%
Gil et al.^49^ and Llorens et al.^51^	Spraying	Vineyard	58%
Gil et al.^50^	Spraying	Vineyard	22%
Maghsoudi et al.^93^	Spraying	Pistachio	34%
Average			40%

## High-resolution 3D modeling of tree crops

**Fig. 3:**
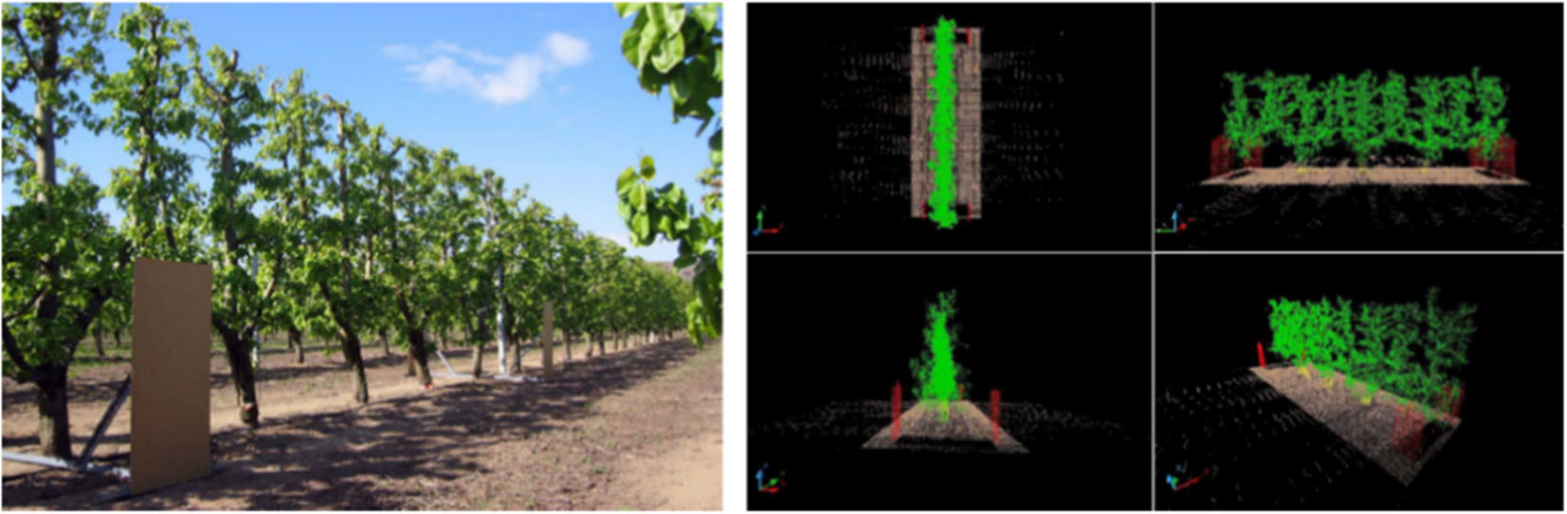
3D point cloud generated by a mobile terrestrial laser scanner in a pear orchard; adapted from Rosell-Polo et al.^53^

Once the variable-rate control and automation was fairly solved and as the LiDAR technology gained attention from researchers^5^, a new study line appeared (at least in terms of horticultural applications) towards the accurate 3D characterization of tree canopies. The approach based on 3D point clouds and 3D modeling from mobile terrestrial laser scanning (MTLS) systems was described in Catalonia by Rosell-Polo et al.^53^ (Fig. 3). Notice that previously discussed studies adopted simpler data visualization and processing through the use of 2D distance images or of simple geometric representations of the scanned target (Fig. 2). As long as appropriate software was used (computer aid design—CAD or similar), this new approach permitted actual 3D visualization and manipulation of the laser data; the point cloud represents all the laser impacts from LiDAR scanning. Given its higher accuracy to represent the tree canopy structures, such 3D models were considered to be applicable not only for variable rate applications (as previously discussed) but also for any application that requires canopy structure characterization in the context of plant phenomics.

The capability of generating 3D models of tree crops from LiDAR scanning using the point cloud approach was tested in laboratory^54–56^, in field conditions^57–60^, and also virtually through simulation software^61–63^. Point cloud approaches were used to identify individual plants in orange groves^58,64^ and apple orchards^65^ enabling tree inventory and in a mango orchard to aid single tree yield estimation^66^. Detailed 3D models were also used to extract woody structures of a pear tree^67^ which can be useful for both plant phenotyping and field applications (e.g., pruning and similar operations). 3D point cloud from LiDAR, coupled with thermal imaging data, was also used in an avocado orchard to access plant physiological status^68^.

**Fig. 4:**
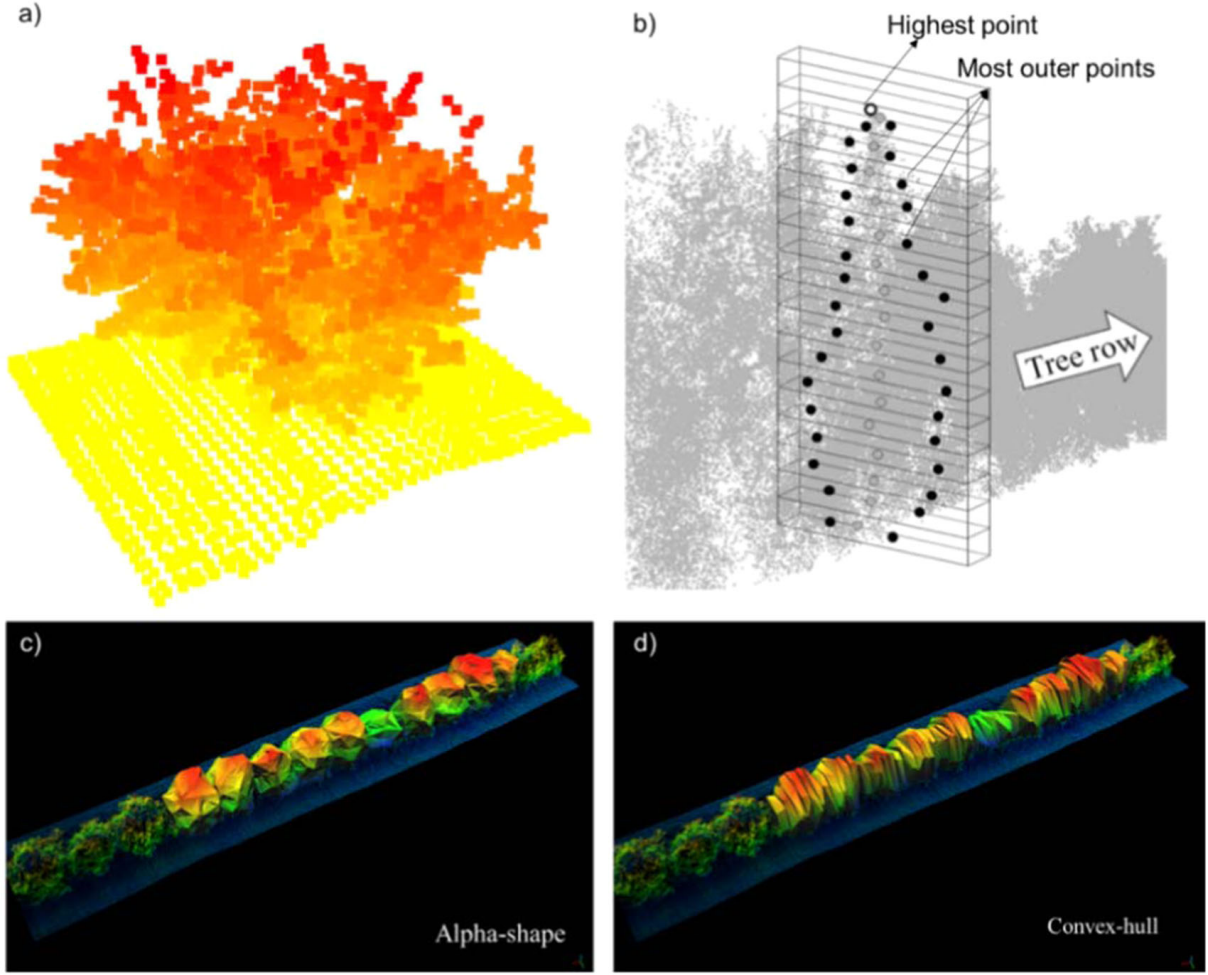
Canopy volume estimation from 3D point clouds. Examples of discretization-based methods using cubes (**a**)^59^ or prisms (**b**)^57^ and by surface reconstruction algorithms (**c** and **d**)^58^

However, the use of point clouds derived from LiDAR scanning would not achieve its purpose for both site-specific management and plant phenotyping unless relevant agronomical parameters are retrieved from it. The canopy volume is one of the most important and studied parameters that can be derived from 3D modeling. In order to compute canopy volume, two main approaches have been reported. The first one is a discretization-based method, which creates a grid of small regular geometries (e.g., cubes or prisms) inside the point cloud structure (Fig. 4a, b). The total volume is obtained by adding up the volumes of such objects. This method is often referred as a “voxel-based” or “occupancy grid.” Usually cubes of equal volume are created inside the point cloud but only those occupied are considered^59,69^. A different “ occupancy” approach was used by Escolà et al.^57^ who applied a method where horizontal prisms of different lengths (equivalent to the canopy width) were stacked in vertical sections of the tree row to calculate the canopy width and subsequently calculate the canopy volume of olive trees.

The second approach is a 3D surface reconstruction, which usually employs triangulation algorithms to connect the outer points of the cloud and create the shape and retrieve the volume of the represented object (Fig. 4c, d). Surface reconstructions based on enclosing objects (hull-based approach) were reported for orange^58^, olive^70^, pear, and apple trees^71^. Auat Cheein and Guivant^72^ applied both the segmented convex hull and the occupancy grid approaches in a point cloud from four pear trees and over a virtual template object. Although a few drawbacks were pointed out, both approaches proved to be effective on characterizing the tree canopies.

Besides canopy volume, algorithms for extracting tree height, width, leaf area index (LAI), and other parameters were reported for different tree crops. Sanz et al.^73^ reported that leaf area was well correlated with canopy volume calculated from LiDAR in apple, pears, and vineyard. The use of the number of impacts of the laser beam also showed potential to estimate leaf area^74^; a strong correlation (*R*^2^ = 0.89) was found between the two parameters. The LAI, which is one of the most widely used indices to characterize grapevines vigor, was well estimated using the TAI (tree area index) from LiDAR scanning (*R*^2^ = 0.91, in Rosell-Polo et al.^71^; and *R*^2^ = 0.92, in Arnó et al^75^. The TAI, similar to the approach proposed by Walklate et al.^26^, uses the probability of the laser beam transmission through the canopy to estimate leaf density. Pforte et al.^76^ compared the leaf area results from the laser scanning with a NIR (near-infrared) image analysis. Correlations between canopy coverage and the leaf area using the LiDAR system yielded better results (*R*^2^ = 0.86) than the image approach.

Other studies focused not necessarily on the data processing but on the different systems and methods used to collect them. Arnó et al.^77^ evaluated the influence of the scanned side of the row in vineyards. They found that, for mapping purposes, the LAI could be estimated from only one side of the row, increasing the efficiency of field operation. The specific row length for accurately estimating LAI from MTLS was also recommended by Arnó et al.^78^. According to Del-Moral-Martínez et al.^79^, in order to produce a reliable map of LAI in vineyards, these measurements do not need to be taken continuously. The discontinuous use of MTLS following a specific sampling scheme is a viable option to overcome difficulties of dealing with large amounts of data from the laser sensor.

Regarding the use of GNSS (Global Navigation Satellite System) in laser scanning, besides enabling the mapping of the measured parameter it solves many problems related with the error of the laser beam positioning from readings taken without a GNSS reference. As shown in Fig. 2, the estimation of the canopy boundaries in early studies, by either laser or ultrasonic measurements, was usually given based on the distance of the sensor from the central line of the row. In other words, the sensor should be kept at a constant known distance from the longitudinal row axis. Therefore, the vehicle should move along the alleyway in a perfect parallel track from the row axis. When plotting a point cloud from a LiDAR scanning in such acquisition systems, all points have a relative position to the centre of the sensor. Therefore, any deviation of the vehicle from the centreline of the alley is transmitted to the 3D positioning of the laser impacts resulting in errors on the final estimated parameter of the trees^47,80^. When a high-accuracy GNSS receiver is attached and synchronized with the acquisition system, the position of the laser impacts can be given relatively to a real geographical positioning of the sensor, and therefore the vehicle does not need to follow a predefined track. Besides, the geographical positioning of the point cloud permits the matching of the two scanned sides of the rows because the points follow the same positioning reference. Figure 3 shows a point cloud from two independent non-georeferenced scanning of a pear grove, one for each side of the row. Because the point cloud was not georeferenced, carton boards (represented in red in the picture) were used as references to manually match the two point clouds from each side. Del-Moral-Martínez et al.^81^ gave a detailed description on how to attribute the GNSS coordinates to each impact of the laser.

Whilst GNSS brings beneficial features to the point cloud and 3D modeling, some drawbacks were pointed by Auat Cheein and Guivant^70^ and Underwood et al.^82^ concerning the limitation of satellite signal in some canopied environments. The authors mention the simultaneous localization and mapping as a non-GNSS-based technique that could overcome positioning issues in such situations. This is particularly important when positioning is used not only to register LiDAR sensor readings but also as a main component of autonomous robotic devices that might be undertaking the scanning.

Another device that can minimize positioning errors of the laser impacts is an inertial measurement unit (IMU). Irregularities of the terrain during the scanning can cause deviations of the angular orientation of the sensor^80^. The IMU can help correcting such positioning error. In the early investigation of LiDAR scanning in tree crops, the laser sensor was commonly used without any additional sensor. Later on, the use of IMUs was reported in some studies^47^ and, most recently, the use of both IMU and high-accuracy GNSS positioning was reported^3,79,81^. Problems with modeling error are not restricted to the agricultural domain. Underwood et al.^83^ gave a detailed description of the different coordinate frames (e.g., the navigation on the environment and the sensor and platform rotations) that must be considered and calibrated when modeling environments (of any kind) using LiDAR platforms.

## Discussions, future challenges, and perspectives

The investigated topics found in the reviewed material were categorized into two main research lines. The first one was characterized by the development and evaluation of sprayers (and some fertilizer spreaders) equipped with either ultrasonic or LiDAR sensors and automatic control of sprayed flow rate. The second research line was characterized by the development of high-resolution 3D modeling by MTLS including aspects of the data acquisition, data processing, and estimation of different tree parameters. 3D modeling has also been the focus of other technologies that were not covered in the present review, but are worth mentioning due to increasing interest by researchers: (i) stereophotogrammetry approaches based on the use of digital RGB cameras and/or multispectral cameras^84,85^, and (ii) active systems based on the use of Depth cameras (also called RGB-D cameras), such as Microsoft’s Kinect^86,87^. The main advantage of LiDAR sensors over passive sensors to collect depth information is the fact that its electromagnetic signal (the laser beam) can penetrate the vegetation canopy, and hence giving information of canopy density and of the inner structures of the trees^1^. Image-based techniques can only access information from the most outer and unobstructed parts of the vegetation. Moreover, image-based techniques are much more affected by the changing outdoors lighting conditions than LiDAR systems. Even being an active sensor, ultrasonic systems have also a limited penetration capability as a result of the great cross-section of the ultrasound beams thus providing measurements with low spatial resolution. It is worth mentioning that ultrasonic sensors have slowly become an outdated technology in digital horticulture as more advanced alternatives become more accurate and accessible. Besides, because they do not have scanning capabilities, ultrasonic systems provide significantly less data than LiDAR or other image-based techniques, which helps explain why such sensors have become less attractive.

From a general assessment over the analyzed studies, it is clear that the major research focus has been on technology development; all studies on both machine automation and 3D modeling were based on self-developed prototypes. Conversely, the application of such technology and its impacts on horticultural research and on the cropping system itself have not been explored at its full potential or made entirely explicit in many studies. Having a clear view of applications and their potential benefit should guide research efforts in terms of required accuracy of information, spatial resolution, and therefore the choice of instrument and data acquisition method. In other words, the level of accuracy and resolution of data must be justified in terms of actual improving diagnostics and management. As an illustration, a range of point cloud densities has been reported (700 points m^−2^ in orange grove by Colaço et al.^58^; 8,800 points m^−2^ in olive grove by Escolà et al.^57^; and over 100,000 points per olive tree by Moorthy et al.^60^; however, the impact of such point densities on the management decisions (e.g., the required amount of inputs) or on the estimation of canopy parameters has not been approached by research yet.

Within the scope of precision horticulture, little effort is noticed on applications other than the real-time variable-rate application of inputs. Ideally, information provided by canopy sensors should be fed into a database gathering other layers of information at the within-grove scale; the work by Mann et al.^88^ is an example of such an approach. Integrating data from ranging sensors with other information such as digital elevation models, soil electrical conductivity, soil fertility, historic yield, and disease and pest occurrence would enable a proper understanding of which factors are driving the tree performance variability, and therefore support an optimized management decision. Such an approach has been common in precision agriculture for broadacre crops, and should be encouraged in precision horticulture research as well. Interpreting the spatial distribution of multiple parameters should help growers recognizing zones with different yield and quality potentials. Even when maps of canopy attributes from ultrasonic or LiDAR sensors were developed^36,40,42,57–59,79,88^, the authors often did not explored the causes of variability or what could be done in terms of management.

The lack of commercial solutions to allow efficient tree characterization might be preventing studies from more practical and holistic agronomic perspectives by researchers who might not have the ability to develop their own scanning equipment. The large amount of published material on different methods of data acquisition and processing should encourage commercial initiatives on providing effective solutions for tree crop scanning.

In order to increase the interest by growers and by other agribusiness players (e.g., the machine, instrumentation, and software industries) on such technology, more studies estimating the potential impact of plant sensing technology to the horticultural industry, the environment and society are encouraged. Reports on input savings from sensor-based variable rate applications are available for different tree crops (Table 1), indicating that such an approach is fairly evolved. However, most of the reported outcomes were based on trials made in small row sections with the main purpose of validating the developed variable-rate prototypes. More comprehensive assessment of the impact of variable-rate technology should be based on whole plot scanning. Besides classic statistical indicators of variability (e.g., histograms, coefficient of variation, etc.), geostatistical analysis should be used in order to characterize plant performance variability at both tree and grove/orchard scales and therefore support estimates about the opportunity for implementing site-specific practices.

In the context of plant phenomics, the reported high-resolution 3D models for different tree crops demonstrate the potential of LiDAR technology for high-throughput phenotyping for aspects related to canopy structure. As highly accurate measurements became possible, the basis of traditional dendrometry methods started to be questioned^89,90^. Early developments of LiDAR-based canopy volume and height estimates were often validated against manual methods used for such measurements^43^. However, as models derived from LiDAR scanning became increasingly more detailed, traditional methods could no longer be used as ground truth measurements, given their low accuracy and subjective measurements^58^. According to the study of Colaço et al.^58^, canopy volume measurements derived from LiDAR should not be validated using former manual methods not only because such methods were inaccurate but also because they were often measuring different types of canopy volume than the ones derived from LiDAR (see above the different approaches to calculate canopy volume—for example, the tree-row-volume, occupancy grid, and surface reconstruction). If different methods result in different plant parameter estimations, it is clear that new standardized methods based on modern plant phenotyping techniques must be developed and adopted in horticultural science.

From the available research on plant phenomics and ranging sensors it was noticeable (through some simple keyword searching) that when research on LiDAR—and other ranging technologies mentioned above—are developed for grain crops, studies are more often related to plant phenomics^4,91,92^ with little application at the crop management level (e.g., for site-specific management); measurements are usually taken in small experimental plots or individual plants in controlled environment. Conversely, in the scope of fruit and nut crops, such technology is more often related to site-specific practices (precision horticulture) than to plant phenotyping per se; for example, to control variable-rate application of inputs. In other words, research on LiDAR and other sensing technologies with a specific aim to enhance plant phenomics through high-throughput phenotyping has being more common for stand crops and should inspire research for the horticultural sector. Even though the potential of LiDAR sensors for plant phenotyping was well demonstrated, the technology has not been adopted for basic horticultural research purposes such as to evaluate the performance of new plant varieties or the outcomes from different management practices. Recently, private and public enterprises started to adopt high-throughput plant phenotyping to aid breeding new grain crop varieties. Commercial solutions for scanning groves, orchards, and nurseries would facilitate adoption in horticulture as well.

In summary, ultrasonic and mostly LiDAR sensors have been regarded as effective tools to collect information about geometrical and structural attributes of tree crops that can be used for high-throughput phenotyping and precision horticulture. During the past two decades, this topic has been investigated by different research groups around the world. Early studies started with the application of ultrasonic sensors. Researchers later turned to the use of LiDAR sensors usually mounted on mobile terrestrial platforms, which showed to be more accurate on the representation of the trees. Fertilizer spreaders and sprayers were equipped with ultrasonic and LiDAR sensors to perform real-time variable rate applications, where inputs were delivered in accordance to the tree size. Studies reported input savings of around 40% by such technology.

Many studies regarding the use of LiDAR sensors in tree crops focused on aspects of data acquisition and mostly data processing. The representation of the trees from LiDAR data evolved to detailed 3D models from which several attributes could be retrieved (e.g., canopy volume, foliage density, and foliage coverage).

As the relevance of such technology was well demonstrated, some aspects of its applicability to precision horticulture are still not been fully explored by research. We believe that research has reached the point where information from sensors should be integrated with other sources of information collected at the within-grove scale, so that the causes of tree performance variability could be identified and managed.

LiDAR and other ranging sensors have shown to be a valuable technology for both plant phenomics and site-specific management, yet they have not been greatly spread amongst researchers and growers. All tools for data acquisition, data processing, and field applications reported in this review were based on research prototypes. Commercial solutions for tree scanning are needed to promote the use and testing of technology by practitioners and academics.

## Footnotes

1 Lidar sensors use a light beam with a relatively small foot-print (some few centimetres according to the model and the distance to the target), while ultrasonic sensors use a cone of ultrasonic waves with higher divergence angle than light beams (usually >5° which turn into some decimeters of foot-print). That gives some laser beams the capacity to penetrate or even trespass the canopy.
